# Successful treatment of type I cryoglobulinemia with a combination of carfilzomib, cyclophosphamide, and dexamethasone: a case report and literature review

**DOI:** 10.1007/s12185-024-03911-z

**Published:** 2025-02-04

**Authors:** Yohei Yasuda, Hiroaki Maki, Arika Shimura, Akira Honda, Yosuke Masamoto, Mineo Kurokawa

**Affiliations:** 1 Department of Hematology and Oncology, Graduate School of Medicine, The University of Tokyo, 7-3-1 Hongo, Bunkyo-Ku, Tokyo, 113-8655 Japan; 2 Department of Cell Therapy and Transplantation Medicine, The University of Tokyo Hospital, 7-3-1 Hongo, Bunkyo-Ku, Tokyo, 113-8655 Japan

**Keywords:** Type I cryoglobulinemia, Plasma cell neoplasm, Carfilzomib, Cyclophosphamide

## Abstract

Type I cryoglobulinemia is typically associated with hematological malignancies such as B-cell lymphomas and plasma cell neoplasms. Its treatment basically targets underlying hematological malignancies, and the prognosis remains unsatisfactory. Despite several reports of type I cryoglobulinemia treated with bortezomib-based regimens, little is available on the treatment of bortezomib-resistant cases. We report a case of severe type I cryoglobulinemia associated with plasma cell neoplasm, refractory to bortezomib and daratumumab, which was successfully managed with a combination of carfilzomib, cyclophosphamide, and dexamethasone (KCd therapy). No sign of relapse has been seen for more than 3 years with maintenance therapy with ongoing carfilzomib. This case highlights the potential efficacy of carfilzomib-based regimens in bortezomib-resistant type I cryoglobulinemia, offering a promising option for cases refractory to conventional treatments.

## Introduction

Cryoglobulinemia is characterized by specific antibodies in the blood that aggregate or precipitate at low (< 37 ℃) temperatures, leading to inflammation and damage in small blood vessels [1–3]. Type II and type III cryoglobulinemia, where cryoglobulins are composed of polyclonal immunoglobulins (typically IgG) with or without monoclonal immunoglobulins (typically IgM with rheumatoid factor activity), are mainly caused by inflammatory conditions, with chronic hepatitis C virus (HCV) infection being the most common, followed by other infectious diseases and autoimmune disorders [4, 5]. In type I cryoglobulinemia, on the other hand, cryoglobulins are monoclonal immunoglobulins basically produced by hematological malignancies, including B-cell lymphomas such as lymphoplasmacytic lymphoma/Waldenström’s macroglobulinemia (20–33%), as well as plasma cell neoplasms such as multiple myeloma (11–20%) and monoclonal gammopathy of undetermined significance (31–44%) [6–10]. Symptoms of type I cryoglobulinemia are primarily related to vessel obstruction and inflammation, presenting as skin lesions (28–86%) with necrosis occurring frequently (10–28%), Raynaud’s syndrome (13–31%), joint pain (13–28%), kidney involvement (14–35%), and peripheral neuropathy (14–44%) [6–10].

While the treatment of cryoglobulinemia basically targets underlying hematological malignancies, the prognosis of type I cryoglobulinemia remains poor. According to the data from the past five cohorts, the 10-year overall survival rates ranged from 52.5 to 68%, except for the study by Terrier et al. that reported a higher rate of 87% [6–10].

Cohort studies and case reports indicate that bortezomib-based regimens have been used for type I cryoglobulinemia associated with plasma cell tumors [6–12]. However, the literature on the treatment of bortezomib-resistant cases is scarce. Herein, we report a case of bortezomib-resistant type I cryoglobulinemia related to plasma cell neoplasm successfully treated with a regimen comprising carfilzomib, cyclophosphamide, and dexamethasone (KCd therapy) [13–15].

## Case presentation

**Fig. 1:**
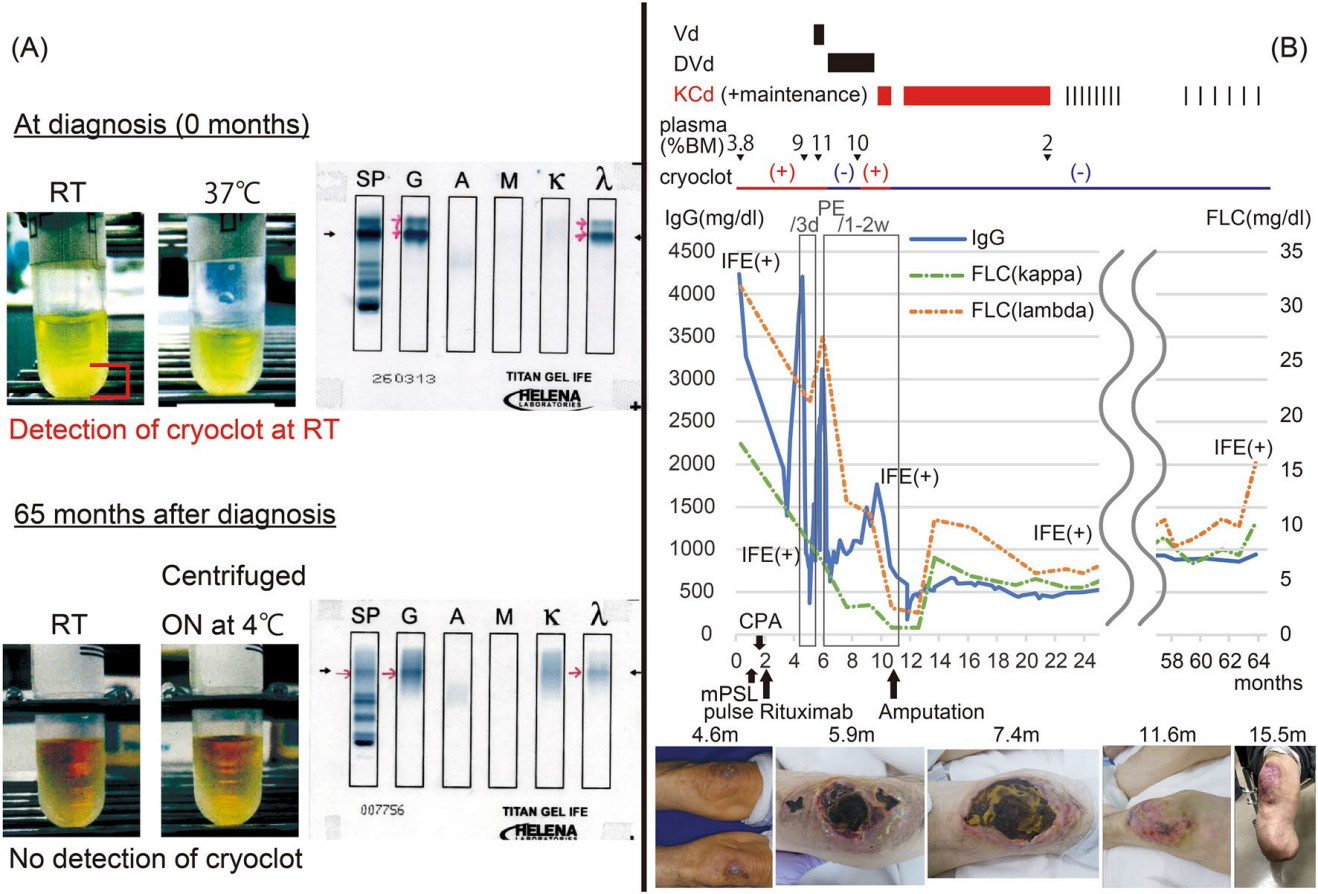
A comparative visualization of cryoclots and serum immunofixation electrophoresis at diagnosis and post-treatment. B Timeline of the clinical progression and treatment interventions. *IFE* immunofixation electrophoresis, *PE* plasma exchange, *FLC* free light chain

A 77-year-old man presented to an outpatient dermatology clinic primarily concerned with skin rashes that persisted for about a year, mainly on his knees, feet, and hands. Some lesions on his feet exhibited minor skin changes suggestive of ischemia. Skin biopsies were performed from a purpura on the feet and an erythema on the hand, revealing numerous fibrin thrombi within small vessels and intravascular inflammatory findings, indicative of leukocytoclastic vasculitis. Regarding vascular risk factors, the patient was an ex-smoker with a history of 20 pack-years and had no history of hypertension, diabetes, or hyperlipidemia. Blood tests showed an elevated IgG level (4208 mg/dl) with other immunoglobulin classes near the lower limits of normal, along with cryoglobulinemia (Fig. 1a). Autoimmune and viral serologies, including hepatitis C, were negative. Immunofixation electrophoresis (IFE) identified two bands of IgG-λ type, indicating aggregation of cryoglobulin (Fig. 1a). The bone marrow examination revealed 3.8% of plasma cells with CD38 + , CD19−, and light chain restriction to lambda, suggesting an abnormal plasma cell population. These findings indicated type I cryoglobulinemia. Upon PET-CT evaluation, no evidence of lymphadenopathy, hepatic or splenic involvement, or other extramedullary manifestations was detected.

Immunosuppressive therapies, including methylprednisolone pulse therapy (1000 mg/day, 3 days) followed by oral prednisone, high-dose cyclophosphamide (500 mg/m^2^), and weekly rituximab (375 mg/m^2^) for 4 weeks, were initially applied by the Dermatology department, ending in a modest reduction in the IgG level to 2005 mg/dl.

A few months later, the patient was emergently hospitalized due to a high fever and subsequently transferred to our department. There was no clinical, laboratory, or imaging evidence of infection. Severe progression of skin necrosis suggested exacerbation of cryoglobulinemia-associated vasculitis. Re-elevation of the IgG level (4,238 mg/dl) prompted the initiation of plasma exchange therapy to reduce cryoglobulins. The bone marrow examination revealed 10% CD138-positive plasma cells in the marrow clot section, while the smear showed 9%. These findings fulfill the diagnostic criteria for multiple myeloma, with anemia serving as a myeloma-defining event.

As causative therapy, we initiated Vd therapy with doses adjusted for frailty comprising bortezomib (1.0 mg/m^2^) and dexamethasone (20 mg/body) on days 1, 8, and 15 of a 28-day cycle [16–18]. The use of immunomodulatory drugs was precluded by deep vein thrombosis detected on ultrasound examination. Despite the initiation of this therapy, there was a gradual increase in IgG levels. Bone marrow aspiration performed 20 days after treatment initiation revealed 11% plasma cells, indicating a lack of response to the therapy. We added weekly daratumumab (16 mg/kg), transitioning to DVd therapy [19] and escalated bortezomib to 1.3 mg/m^2^ to intensify treatment, while maintaining a 28-day cycle. Although the regimen moderately reduced serum IgG levels and temporarily made cryoclots undetectable during the first two cycles, a recurrence in IgG levels and the reappearance of cryoclots was observed during the subsequent two cycles. The progression of skin necrosis, particularly in the knees and feet, persisted, leading us to prepare for above-knee amputation of both legs. We altered the regimen to KCd therapy in unfit doses, comprising carfilzomib (36 mg/m^2^), cyclophosphamide (150 mg/m^2^) administered on days 1, 8, and 15 of a 28-day cycle with weekly dexamethasone (20 mg/body) [13–15, 18]. Cyclophosphamide was included to enhance therapeutic efficacy, as dose escalation of carfilzomib was limited by frailty and immunomodulatory drugs were contraindicated due to persistent deep vein thrombosis. It quickly decreased serum IgG levels and eliminated cryoglobulins, enabling discontinuation of plasma exchange therapy. The ischemic lesions showed marked improvement. The necrosis in the lesions below the ankles had already progressed to an irreversible stage, still requiring amputation. However, as the blood flow in the knees had improved, the amputation was performed below the knees on both lower limbs. As a representative indicator of the necrotic lesions, the time-series images of the knee lesions are illustrated in the clinical chart (Fig. 1b).

Nine cycles in total were completed without severe non-hematological toxicity. With the subsequent ongoing carfilzomib maintenance, no progression of symptoms or re-emergence of cryoclots has been detected for over 3 years, while IFE remained positive for IgG-λ throughout the entire clinical course.

## Discussion

In the present case, KCd (carfilzomib, cyclophosphamide, and dexamethasone) therapy achieved rapid and long-lasting remission in a patient with bortezomib-refractory type I cryoglobulinemia associated with a plasma cell neoplasm. Even with the inclusion of bortezomib in plasma cell neoplasms cases, the most recent cohort showed that the 5-year event-free survival rate for IgG type I cryoglobulinemia remains low at just 8% [9]. The present case was especially challenging, suggesting a considerably poor prognosis, as the patient showed total refractoriness to initial VD (bortezomib and dexamethasone) therapy. However, the introduction of KCd therapy demonstrated remarkable efficacy in overcoming bortezomib resistance in our case.

One of the most striking features of this case was the rapidity with which cryoglobulins disappeared and symptoms improved. This remarkable outcome suggested strong selectivity of carfilzomib for cryoglobulin-producing clones. The persistent detection of M protein despite the complete elimination of cryoglobulins further supports this hypothesis, considering the heterogeneity of the disease. Carfilzomib, an irreversible and specific inhibitor of chymotrypsin-like activities of proteasomes [20], might particularly challenge these clones with the production of especially abnormal globulins.

This case highlights the difficulties in managing type I cryoglobulinemia. Although immunosuppressive therapies including rituximab are commonly used for symptomatic relief [7], expert opinions recommend prioritizing treatment of underlying malignancies, as mentioned in the introduction section [2]. In our case, the initial approach of immunosuppressive therapy, chosen due to low tumor burden, resulted in rapid progression of vasculitis, reinforcing the importance of proactive initiation of chemotherapy.

The concept of “Monoclonal gammopathy of clinical significance” (MGCS), including type I cryoglobulinemia, has been recently proposed[21], in which even small amounts of monoclonal proteins can cause significant clinical symptoms. The decision to initiate polychemotherapy for underlying malignancies should be promptly determined based on the patient’s symptomatic presentation rather than on quantitative parameters of tumor burden or cryoglobulin levels. In light of this concept, the presented case suggests that progressing necrotic lesions may serve as an indicator for proactive therapeutic intervention. Furthermore, prompt salvage therapies should be considered even in cases where first-line therapies partially improve cryoglobulin levels.

To clarify the result of previous studies on type I cryoglobulinemia, clinical manifestations and the prognoses are summarized in a table (Table 1) [6–10]. The studies share many common findings, notably the high prevalence of MGUS as an underlying disease. This prevalence of MGUS is significant as it suggests that even a small number of tumor cells can lead to the development of the disease. Regarding symptoms, skin lesions are frequently reported, with a notable incidence of necrosis. Moreover, except for the study by Terrier et al., these studies generally report a poor long-term prognosis. The study by Ghembaza et al. analyzed prognosis based on underlying disorders and Ig type, showing inferior outcomes for IgG type, along with higher incidence of necrosis and renal failure [9].

**Table 1:** Summary of major cohorts of type I cryoglobulinemia

Study	Terrier 2013	Harel 2014	Néel 2014	Sidana 2017	Ghembaza 2023
Nation	France	France	France	US	France
Characteristics					
Number of patients	64	64	36	102	168
Median age (years)	65	62	63	59	65
IgG type (%)	N/A	60	31	53	45
Hematological malignancy (%)					
MGUS	44	41	36	38	31
MM	19	19	11	20	15
LPL	20	28	33	21	27
Other B-cell lymphomas	17	13	19	14	7
Symptoms (%)					
Skin manifestations	86	58	58	63	**IgG: 45** IgM: 15
Necrosis	28	**IgG: 37** IgM: 7.7	**IgG: 55** IgM: 8	34	**IgG: 18** IgM: 3
Renal involvement	30	**IgG: 29** IgM: 7.7	22	14	**IgG: 54** IgM: 19
Raynaud syndrome	N/A	19	31	25	13
Joint arthralgia	28	N/A	19	24	13
Peripheral neuropathy	44	14	39	32	20
Prognosis					
5-year PFS (%)	N/A	28	N/A	N/A	**IgG: 8** IgM: 47
5-year OS (%)	94	83	82	N/A	77
10-year OS (%)	87	68	60	Median OS: 11.4 years	**IgG: 36** IgM: 71

Bold values indicate significant differences (*p <* 0.05) between IgG and IgM types

For the Néel [10] and Sidana [7] studies, the ‘Necrosis’ category includes both necrosis and ulcers

*MGUS* monoclonal gammopathy of undetermined significance, *MM* multiple myeloma, *LPL* lymphoplasmacytic lymphoma, *PFS* progression-free survival, *OS* overall survival

Regarding novel therapies including bortezomib and/or immunomodulators, high response rates have been reported, although long-term efficacy remains limited. In the study by Sidana et al., ten of twelve patients experienced symptom relief [7]. For IgG-type cryoglobulinemia, the study by Ghembaza et al. reports complete symptom relief in 6 months in 60–100% of patients. However, the 5-year event-free survival (EFS) rate for IgG-type patients, of whom 39% were treated with bortezomib, was only 8% in the same cohort [9]. Given the persistently high incidence of relapse, it is considered necessary to explore treatment options for relapsed or refractory cases. Nevertheless, studies reporting effective treatments for bortezomib-resistant cases are scarce. It appears crucial to accumulate cases and knowledge regarding salvage therapies, especially those using emerging drugs such as other PIs and molecular targeted drugs such as anti-CD38 monoclonal antibody. The present case provides important insights in this context, despite the limitations of being a single case report with a limited observation period. In addition, the possibility that the dose reduction of bortezomib due to frailty compromised its efficacy should be considered as a limitation in interpreting the resistance to bortezomib.

In conclusion, this case report demonstrates the remarkable efficacy of a carfilzomib-based therapy in achieving rapid and long-lasting remission in bortezomib-refractory type I cryoglobulinemia associated with a plasma cell neoplasm. This finding is particularly significant given the scarcity of reports on effective treatments for bortezomib-resistant cases in the literature. Although further validation through the accumulation of additional cases and large-scale studies is necessary, carfilzomib-based regimens appear to be a promising therapeutic approach for this challenging clinical scenario.

## Data Availability

The data that support the findings of this case report are available upon reasonable request, subject to privacy and ethical restrictions.
